# Investigation of optimal gestational weight gain for twin pregnancy in Southwest China: a retrospective study

**DOI:** 10.1038/s41598-023-31766-7

**Published:** 2023-03-28

**Authors:** Li Gao, Cuirong Lei, Shuwei Zhou, Qianqian Liao, Lingwei Mei, Qimei Zhong, Xia Lan, Ya Chen, Lan Wang

**Affiliations:** 1 Department of Obstetrics and Gynecology, Chongqing Health Center for Women and Children (Women and Children’s Hospital of Chongqing Medical University), Chongqing, 401147 China; 2 Gynecological Oncology Center, Chongqing University Cancer Hospital, Chongqing, 400030 China

**Keywords:** Health care, Medical research

## Abstract

There is a lack of data on gestational weight gain (GWG) in twin pregnancies. We divided all the participants into two subgroups: the optimal outcome subgroup and the adverse outcome subgroup. They were also stratified according to prepregnancy body mass index (BMI): underweight (< 18.5 kg/m^2^), normal weight (18.5–23.9 kg/m^2^), overweight (24–27.9 kg/m^2^), and obese (≥ 28 kg/m^2^). We used 2 steps to confirm the optimal range of GWG. The first step was proposing the optimal range of GWG using a statistical-based method (the interquartile range of GWG in the optimal outcome subgroup). The second step was confirming the proposed optimal range of GWG via compared the incidence of pregnancy complications in groups below or above the optimal GWG and analyzed the relationship between weekly GWG and pregnancy complications to validated the rationality of optimal weekly GWG through logistic regression. The optimal GWG calculated in our study was lower than that recommended by the Institute of Medicine. Except for the obese group, in the other 3 BMI groups, the overall disease incidence within the recommendation was lower than that outside the recommendation. Insufficient weekly GWG increased the risk of gestational diabetes mellitus, premature rupture of membranes, preterm birth and fetal growth restriction. Excessive weekly GWG increased the risk of gestational hypertension and preeclampsia. The association varied with prepregnancy BMI. In conclusion, we provide preliminary Chinese GWG optimal range which derived from twin-pregnant women with optimal outcomes(16–21.5 kg for underweight, 15–21.1 kg for normal weight, 13–20 kg for overweight), except for obesity, due to the limited sample size.

## Introduction

Women carrying inappropriate weight gain are at increased risk of preeclampsia (PE), gestational diabetes mellitus (GDM), preterm birth (PB), fetal growth restriction (FGR), macrosomia, and large-for-gestational-age infants^1–3^. It is well known that women with multiple pregnancies suffer from more adverse pregnancy outcomes than those with singleton pregnancies^4,5^. Twins account for a quarter of low-birth-weight babies, approximately half of all twins are premature, and one-sixth of all neonatal deaths are twins^6–8^. With the widespread use of assisted reproductive technology (ART), an increase of 22.5% in the proportion of twin pregnancies occurred only during the decade between 2005 and 2015^9^, and this number is estimated to increase in the coming years. Therefore, more attention should be givenpaid to twin pregnancy.

Gestational weight gain (GWG) can be an indicator of the nutritional level of pregnant women and is closely associated with maternal and fetal outcomes^10,11^. Combining higher pregnancy risk with increased nutritional demands in twin pregnancies than in singleton pregnancies, optimal weight gain for twin pregnancies deserves more examination^12^. Numerous studies have focused on singleton gestation, and the Institute of Medicine (IOM) provided guidelines for optimal weight gain during pregnancy in 2009, whereas only “provisional” guidelines were provided for twin pregnancy^12,13^. In view of the IOM recommendations, many studies about the association of GWG and pregnancy outcomes have been conducted in twin pregnancy, but very few studies have focused on non-industrialized countries, including China^14^ Due to differences between industrialized countries and non-industrialized countries in ethnicity, culture, and dietary habits, Chinese adult BMI levels are lower than those found in the World Health Organization (WHO), and their recommendations may not be appropriate for Chinese pregnant women^10^. Thus, the aim of our study was to explore an optimal GWG range that is suitable for twin pregnancies in the Chinese population.

## Results

### Patient characteristics and outcomes

**Figure 1:**
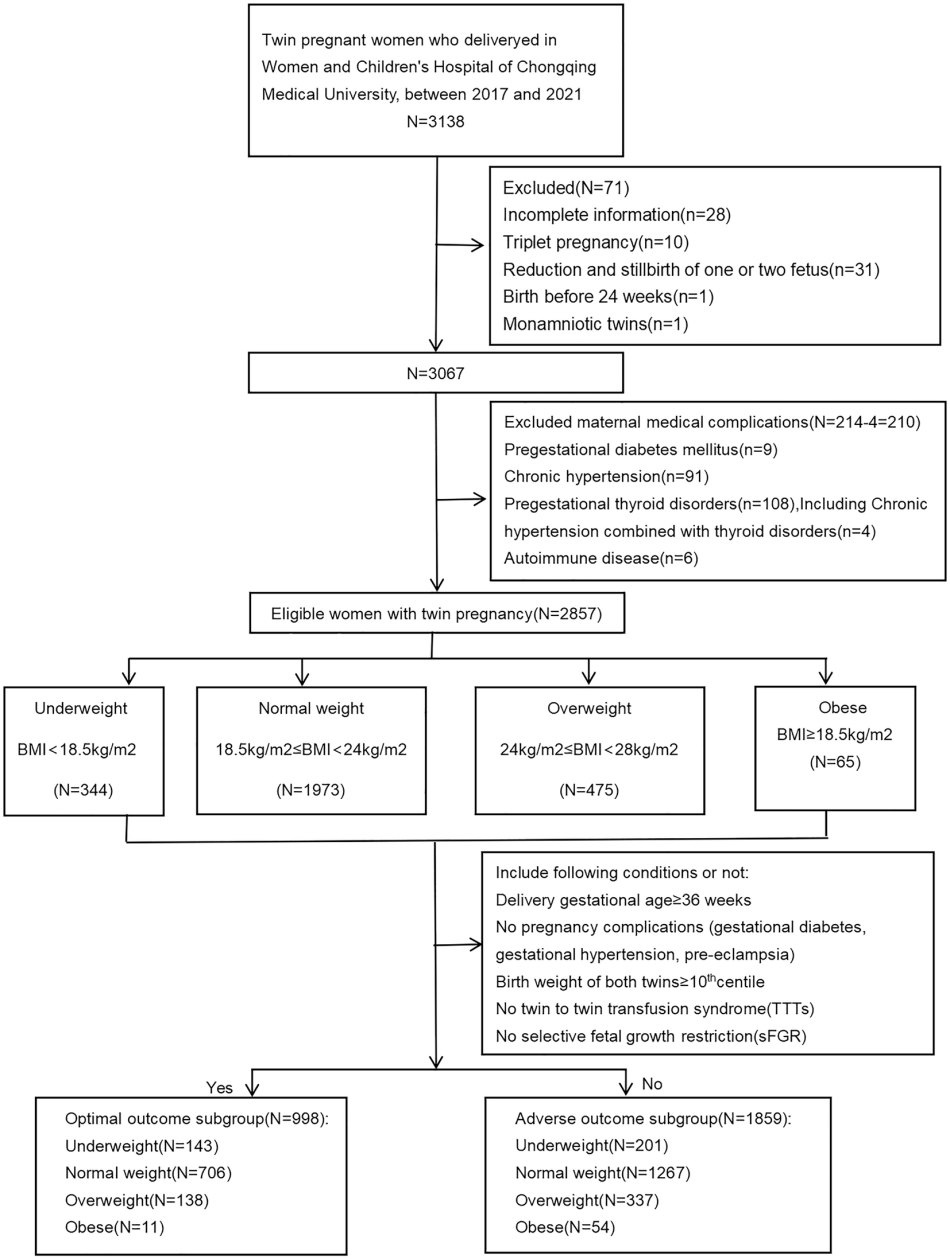
Flowchart of the enrolled participants.

There were 3138 individuals with twin pregnancies diagnosed between January 2017 and December 2021; 2857 met the study criteria: 998 (34.9%) were included in the optimal outcome subgroup (women with no complications and good neonatal outcome), and 1859 (65.1%) were included in the adverse outcome subgroup. According to the maternal prepregnancy BMI, 143 were underweight, 706 were normal weight, 138 were overweight, and only 11 were obese in the optimal outcome subgroup (Fig. 1).

According to the maternal prepregnancy BMI, the basic characteristics of the study cohort are presented in Table 1. A total of 2857 pregnant women were enrolled, of whom 344 (12.0%) were underweight, 1973 (69.1%) were normal weight, 475 (16.6%) were overweight, and 65 (2.3%) were obese. The underweight group had the lowest mean age (29.2 ± 3.7 years, F = 29.453, *P* < 0.001), the lowest prevalence of GDM (16.0%, χ^2^ = 66.707, *P* < 0.001) and PE (11.0%, χ^2^ = 24.156, *P* < 0.001), and the highest prevalence of FGR though there were no statistically difference (7.4%, χ^2^ = 3.602, *P* = 0.308). The number of obese group was the lowest, but the proportion of maternal age older than 35 (27.7%, χ^2^ = 35.935, *P* < 0.001)and ART for pregnancy (76.9%, χ^2^ = 25.389, *P* < 0.001)were the highest as well as pregnancy complications including GH(10.8%, χ^2^ = 10.708, *P* = 0.013), PE (29.2%, χ^2^ = 24.156, *P* < 0.001)and GDM (49.2%, χ^2^ = 66.707, *P* < 0.001, respectively. Moreover, the terminal gestational age of 32–34 weeks (13.8%, χ^2^ = 8.138, *P* = 0.043) and less than 28 weeks (3.1%, χ^2^ = 3.400, *P* = 0.334) also accounted for the highest proportion in the obese group. There were statistically significant differences among the 4 BMI groups with respect to age, nulliparity, past cesarean delivery, ART, GH, PE, GDM and gestational weeks at birth.

**Table 1:** Maternal and neonatal characteristics grouped by prepregnancy BMI.

	Underweight N = 344	Normalweight N = 1973	Overweight N = 475	Obesity N = 65	F/χ2	*P* value
Maternal age (years), mean ± SD	29.2 ± 3.7	30.8 ± 3.7	31.6 ± 4.0	32.4 ± 3.8	29.453	< ***0.001***
Maternal age (years) ≥ 35	25 (7.3)	307 (15.6)	99 (20.8)	18 (27.7)	35.935	< ***0.001***
Nulliparity	287 (83)	1699 (86.1)	378 (79.6)	51 (78.4)	14.831	***0.002***
Dichorionic	268 (78)	1663 (84.2)	417 (87.8)	57 (87.7)	15.430	***0.001***
Monochorionic	71 (21)	304 (15.4)	50 (10.5)	7 (10.8)	17.062	***0.001***
Unknown chorionicity	5 (1.5)	6 (0.3)	8 (1.7)	1 (1.5)	14.535	***0.002***
Past preterm birth	3 (0.9)	5 (0.2)	1 (0.2)	0 (0)	4.009	0.261
Past cesarean delivery	27 (7.8)	160 (8.1)	65 (13.7)	15 (23.1)	29.488	< ***0.001***
Assisted reproductive technology (ART)	207 (60.2)	1379 (69.9)	361 (76)	50 (76.9)	25.389	< ***0.001***
Gestational diabetes mellitus(GDM)	55 (16)	496 (25.1)	177 (37.2)	32 (49.2)	66.707	< ***0.001***
Gestational hypertension (GH)	16 (4.7)	68 (3.4)	15 (3.2)	7 (10.8)	10.708	***0.013***
Preeclampsia (PE)	38 (11)	279 (14.1)	94 (19.8)	19 (29.2)	24.156	< ***0.001***
Fetal growth restriction(FGR)	26 (7.4)	119 (6.0)	21 (4.4)	4 (6.2)	3.602	0.308
Premature rupture of membranes (PROM)	71 (20.1)	398 (20.2)	86 (18.1)	13 (20)	1.170	0.760
Gestational weeks(w) at birth						
≥ 36w	213 (62)	1271 (64.4)	313 (65.9)	32 (49.2)	7.693	0.053
34–36w	69 (20)	434 (22)	89 (18.7)	19 (29.2)	5.161	0.160
32–34w	39 (11)	153 (7.8)	35 (7.3)	9 (13.8)	8.138	***0.043***
28–32w	16 (4.7)	88 (4.5)	27 (5.7)	3 (4.6)	1.285	0.733
< 28w	7 (2)	27 (1.4)	11 (2.3)	2 (3.1)	3.400	0.334

*P* < 0.05 was considered statistically significant.

Significant values are in [bolditalics].

### The first step was proposing the optimal GWG range: a statistical-based approach

**Figure 2:**
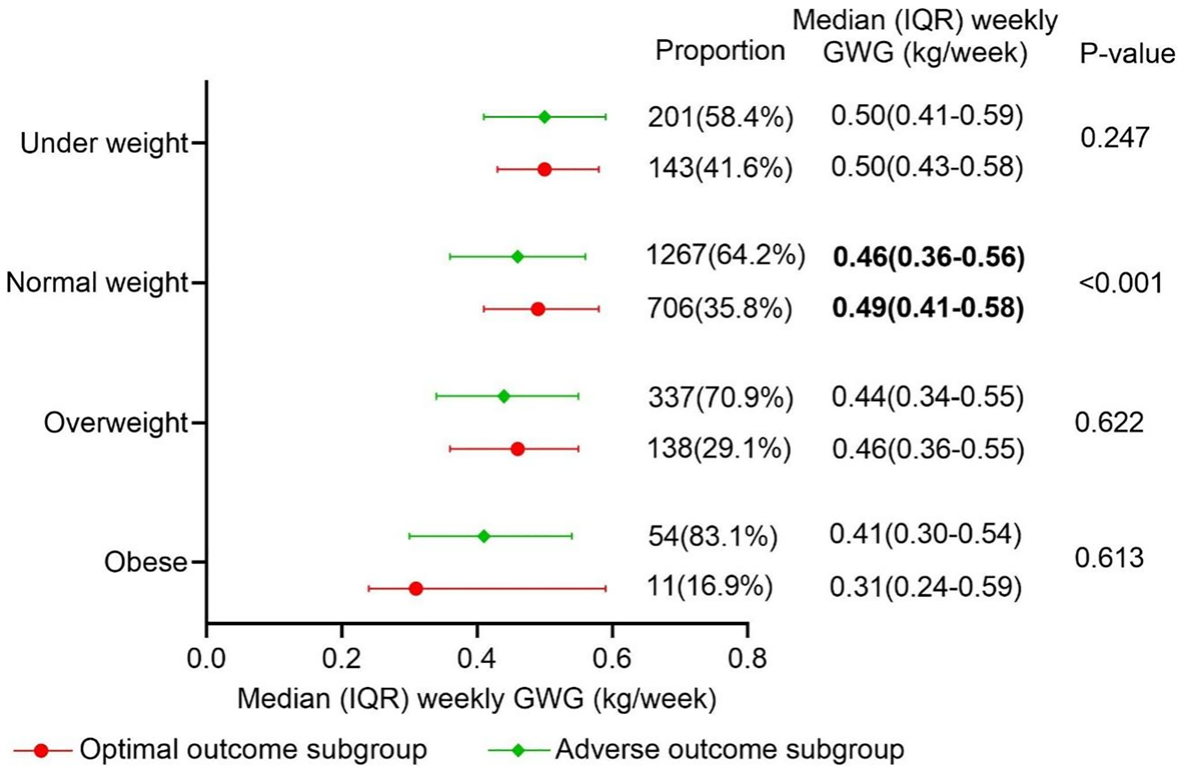
Distribution of weekly gestational weight gain in the optimal- and adverse-outcome subgroups. *P* < 0.05 was considered statistically significant. GWG gestational weight gain; IQR interquartile range.

To determine the optimal GWG range, we used the interquartile range to obtain a range of GWG and weekly GWG in the optimal outcome subgroup. The number of normal weight accounted for 70.7% of the total (706/998). An optimal GWG of 16–21.5 kg was obtained in the underweight group, 15–21.1 kg was obtained in the normal weight group, 13–20 kg was obtained in the overweight group, and 9–22 kg was obtained in the obese group (Table 2). Except for the obese group, the optimal GWG was lower than the IOM recommendations, which were recommended to be 16.8–24.5 kg, 14.1–22.7 kg, and 11.3–19.1 kg for normal weight, overweight, and obese, respectively (Table 3). Due to the different gestational weeks at birth, we calculated the distribution of weekly GWG (in kg/wk) in the 4 BMI groups (Table 2). and compared the weekly GWG between the optimal outcome subgroup and the adverse outcome subgroup (Fig. 2). In normal weight and overweight women, the optimal outcome subgroup had a higher median weekly GWG than the adverse outcome subgroup, whereas in obese women, the optimal outcome subgroup had a lower median weekly GWG than the adverse outcome subgroup, and a statistically significant difference for median weekly GWG was only found in normal weight women (*p* < 0.001) (Fig. 2).

**Table 2:** This distribution of optimal total GWG and weekly GWG in the optimal outcome subgroup.

	BMI standard (kg/m^2^)	No. of subjects (%)	Median of optimal total GWG(kg)	IQR of optimal total GWG(kg)	Median of optimal weekly GWG(kg/w)	IQR of optimal weekly GWG (kg/w)
Underweight	< 18.5	143 (14.3)	18.5	16–21.5	0.50	0.43–0.58
Normalweight	18.5 ≤ BMI < 24	706 (70.7)	18	15–21.1	0.49	0.41–0.58
Overweight	24 ≤ BMI < 28	138 (13.8)	16.8	13–20	0.46	0.35–0.55
Obese	≥ 28	11 (1.1)	11	9–22	0.31	0.24–0.59

*GWG* gestational weight gain, *BMI* body mass index, *IQR* interquartile range.

**Table 3:** The comparison between proposed optimal GWG and IOM recommended GWG grouped by prepregnancy BMI.

	Chinese BMI standard(kg/m^2^)	Optimal total GWG(kg)	IOM BMI standard(kg/m^2^)	IOM recommended GWG(kg)	IOM recommended weekly GWG(kg/w)
Underweight	< 18.5	16–21.5	< 18.5	–	–
Normal weight	18.5 ≤ BMI < 24	15–21.1	18.5 ≤ BMI < 25	16.8–24.5	0.45–0.66
Overweight	24 ≤ BMI < 28	13–20	25 ≤ BMI < 30	14.1–22.7	0.38–0.61
Obese	≥ 28	9–22	≥ 30	11.3–19.1	0.31–0.52

*BMI* body mass index, *GWG* gestational weight gain, *IOM* Institute of Medicine.

### The second step was confirming the proposed optimal GWG range: an outcome-based approach

To further verify the rationality of the proposed GWG optimal range, we compared the incidence of pregnancy complications within and outside the weekly GWG optimal range and explored the relationship between weekly GWG and complications through logistic regression analysis (Tables 4, 5). A represents the group within the recommendation, B represents the group outside the GWG optimal range (including below and above the recommendations), C represents the group below the GWG optimal range, and D represents the group above the GWG optimal range.

**Table 4:** The incidence of pregnancy complications in the population below or above the optimal GWG was compared.

	A: weeklyGWG:0.43–0.58 (167)	B: weekly GWG < 0.43 or > 0.58 (177)	C: weekly GWG < 0.43 (86)	D: weekly GWG > 0.50 8 (91)	P: A vs B	P: C vs D	P : A vs C	P : A vs D
Underweight (344)								
GH	9 (5.4%)	7 (4.0%)	1 (1.2%)	6 (6.6%)	0.528	0.064	0.102	0.693
PE	18 (10.8%)	18 (10.2%)	6 (7.0%)	12 (13.2%)	0.854	0.172	0.328	0.564
GDM	19 (11.4%)	36 (20.3%)	24 (27.9%)	12 (13.2%)	***0.016***	***0.015***	***0.001***	0.167
PROM	34 (20.4%)	37 (20.9%)	24 (27.9%)	13 (14.3%)	0.901	***0.026***	0.176	0.227
PB < 36w	58 (34.7%)	73 (41.2%)	46 (53.5%)	27 (29.7%)	0.060	***0.001***	***0.004***	0.409
FGR	13 (7.8%)	12 (6.8%)	6 (7.0%)	6 (6.6%)	0.720	0.919	0.817	0.726

	A: weeklyGWG:0.41–0.58 (855)	B: weekly GWG < 0.41 or > 0.58 (1118)	C: weekly GWG < 0.41 (673)	D: weekly GWG > 0.58 (445)	P: A vs B	P: C vs D	P: A vs C	P: A vs D
Normal weight (1973)								
GH	27 (3.2%)	41 (3.4%)	18 (2.8%)	23 (5.2%)	0.539	***0.030***	0.579	0.074
PE	120 (14.0%)	159 (14.2%)	52 (7.7%)	107 (24.0%)	0.906	** < *0.001***	** < *0.001***	** < *0.001***
GDM	183 (21.4%)	313 (28.0%)	236 (35.1%)	77 (17.3%)	0.001	** < *0.001***	** < *0.001***	0.079
PROM	165 (19.4%)	233 (20.8%)	161 (23.9%)	72 (16.2%)	0.397	***0.001***	***0.028***	0.167
PB < 36w	308 (36.0%)	394 (35.2%)	279 (41.5%)	115 (25.8%)	0.719	** < *0.001***	***0.030***	** < *0.001***
FGR	49 (5.7%)	70 (6.3%)	47 (7.0%)	23 (5.2%)	0.624	0.188	0.316	0.674

	A: weeklyGWG: 0.36–0.55 (219)	B: weekly GWG < 0.36 or > 0.55 (256)	C: weekly GWG < 0.36 (135)	D: weekly GWG > 0.55 (121)	P: A vs B	P: C vs D	P: A vs C	P: A vs D
Over weight (475)								
GH	5 (2.3%)	10 (3.9%)	5 (3.7%)	5 (4.1%)	0.313	0.860	0.433	0.334
PE	40 (18.3%)	54 (21.1%)	14 (10.4%)	40 (33.1%)	0.440	< ***0.001***	***0.045***	***0.002***
GDM	78 (35.6%)	99 (30.5%)	63 (46.7%)	36 (29.8%)	0.556	0.669	***0.039***	0.273
PROM	32 (14.6%)	54 (21.1%)	31 (23.0%)	23 (19.0%)	0.067	0.439	***0.046***	0.309
PB < 36w	34 (15.5%)	41 (16.0%)	23 (17.0%)	18 (14.9%)	0.884	0.638	0.707	0.847
FGR	11 (5.0%)	10 (3.9%)	7 (5.2%)	3 (2.5%)	0.555	0.265	0.946	0.258

	A: weekly GWG:0.24–0.59 (40)	B: weekly GWG < 0.24 or > 0.5 (25)	C: weekly GWG < 0.24 (12)	D: weekly GWG > 0.59 (13)	P: A vs B	P: C vs D	P: A vs C	P: A vs D
Obese (65)								
GH	6 (15.0%)	1 (4.0%)	1 (8.3%)	0 (0.0%)	0.164	0.212	0.553	0.138
PE	13 (32.5%)	6 (25.0%)	0 (0.0%)	6 (46.2%)	0.464	***0.007***	***0.023***	0.372
GDM	22 (55.0%)	10 (40.0%)	6 (50.0%)	4 (30.8%)	0.239	0.327	0.761	0.129
PROM	8 (20.0%)	3 (12.0%)	2 (16.7%)	1 (7.7%)	***0.033***	0.490	0.797	0.305
PB < 36w	22 (55.0%)	11 (44.0%)	7 (58.3%)	4 (30.8%)	0.403	0.165	0.838	0.129
FGR	4 (10.0%)	0 (0.0%)	0 (0.0%)	0 (0.0%)	0.103	–	0.254	0.236

*P* < 0.05 was considered statistically significant.

*GWG* gestational weight gain, *GH* gestational hypertension, *PE* preeclampsia, *GDM* gestational diabetes mellitus, *PROM* premature rupture of membranes, *PB* preterm birth, *FGR* fetal growth restriction. A represents the women in the optimal GWG range. B represents the women outside the optimal GWG range. C represents the women below the optimal GWG range. D represents the women exceed the optimal GWG range.

Significant values are in [bolditalics].

**Table 5:** Multivariable Logistic regression analysis result for weekly GWG impact on pregnancy complications grouped by BMI.

BMI group	factors	OR(95%CI)	*P* value
Underweight	GH	10.84 (0.17–692.848)	0.261
	PE	11.167 (0.665–187.617)	0.094
	GDM	0.096 (0.007–1.363)	0.083
	PROM	0.192 (0.02–1.838)	0.152
	PB** < **36w	0.079 (0.012–0.539)	***0.01***
	FGR	0.891 (0.028–28.828)	0.948
Normal weight	GH	5.497 (1.033–29.249)	0.046
	PE	46.393 (18.78–114.6)	** < *0.001***
	GDM	0.062 (0.028–0.136)	** < *0.001***
	PROM	0.267 (0.121–0.593)	***0.001***
	PB** < **36w	0.125 (0.063–0.246)	** < *0.001***
	FGR	0.375 (0.098–1.443)	0.154
Overweight	GH	1.93 (0.068–55.151)	0.701
	PE	41.593 (8.546–202.421)	** < *0.001***
	GDM	0.178 (0.05–0.625)	***0.007***
	PROM	0.394 (0.084–1.854)	0.238
	PB** < **36w	0.214 (0.03–1.528)	0.124
	FGR	0.21 (0.011–3.939)	0.296
Obese	GH	0.992 (0.016–63.335)	0.997
	PE	38.691 (1.65–907.185)	***0.023***
	GDM	1.301 (0.104–16.229)	0.838
	PROM	0.338 (0.013–8.936)	0.516
	PB** < **36w	0.22 (0.017–2.766)	0.241
	FGR	0.59 (0.003–117.389)	0.845

*P* < 0.05 was considered statistically significant.

*CI* confidence interval, *OR* odds ratio, *GH* gestational hypertension, *PE* preeclampsia, *GDM* gestational diabetes mellitus, *PROM* premature rupture of membranes, *PB* preterm birth, *FGR* fetal growth restriction. Confounding factor including age, height, prepregnancy weight, prepregnancy BMI.

Significant values are in [bolditalics].

From the analysis of Table 4, we can draw the following seven points based on the results: 1, In the underweight, normal weight and obese groups but not overweight group, the incidence of adverse outcomes among women in optimal weekly GWG group was lower than those in outside the optimal range group. 2, Women with insufficient weekly GWG increased the risk of GDM compare to those with optimal weekly GWG in three BMI groups except the obese group. 3, Excessive weekly GWG increased the risk of PE compare to within weekly GWG optimal range, while insufficient weekly GWG decreased the risk of PE compare to with optimal weekly GWG among women in normal weight and overweight groups. 4, Women with insufficient weekly GWG increased the risk of PROM compare to those with optimal weekly GWG in normal weight and overweight groups 5, Compare to women with optimal weekly GWG, the risk of PB < 36w was increased in women with insufficient weekly GWG, whereas the risk of PB < 36w was reduced in women with excessive weekly GWG among normal weight group. 6, Compare to women with optimal weekly GWG, no significant differences were found in the incidence of GH neither women with insufficient weekly GWG nor women with excessive weekly GWG. 7, No significant differences were found between different comparisons in terms of FGR.

From the analysis of Table 5, we can draw the following conclusions: 1, In underweight pregnant women, the incidence of PB was related to weekly GWG (OR 0.079, 95% CI 0.012–0.539, *P* = 0.01). 2, In normal weight pregnant women, the incidence of pregnancy complications (GH, PE, GDM, PB, PROM) was related to weekly GWG (*P* < 0.05), but no relationship was found between FGR and weekly GWG (OR 0.375, 95% CI 0.098–1.443, *P* = 0.154). 3, In overweight pregnant women, the incidence of PE and GDM were related to weekly GWG (*P* < 0.05). 4, In obese pregnant women, the incidence of PE was related to weekly GWG (OR 38.691, 95% CI 1.65–907.185, *P* = 0.023).

## Discussion

Because there are no guidelines for GWG in twin pregnancies, especially poor studies conducted in the Chinese population, the aim of this study is to probe the optimal GWG range in Chinese twin populations, and the reliability of optimal GWG was further validated by the association between inappropriate GWG and adverse outcomes.

The GWG optimal range which derives from the statistical-based approach in the current study is lower than the IOM recommendation and the recommendation proposed by Yawen Chen et al^14,15^. The differences with IOM are most likely due to environmental, dietary, and ethnic differences and sample size. Yawen Chen studied 6925 twin pregnant women who gave birth in Wuhan from 2011 to 2017 and obtained the recommendations of GWG, which is 18–26 kg for underweight(16–21.5 kg in our study), 15–25 kg for normal weight(15–21.1 kg in our study), 12–21 kg for overweight(13–20 kg in our study), and 9–20 kg for obese(9–22 kg in our study)^14^. Although the sample size of the GWG given by Yawen Chen was larger than that of our study, Yawen Chen did not divide the population with good outcomes to confirm optimal GWG, which may be the main reason for the difference between our study. Additionally, the upper limit of the GWG (9–22 kg) in the obese group of our recommendation is higher than the IOM recommendation, which may be caused by the very small number of cases in our obese group, only 11 cases. Due to the limited sample size, the results of the obese group may be limited sense of meaning, not as a recommendation.

To justify our GWG optimal range, we compared the risk of pregnancy complications in 4 BMI groups within and outside the GWG optimal range in a total of 2857 twin pregnancies and finally proved that the risks of disease within the GWG optimal range were lower than those outside the GWG optimal range. GH and PE are associated with weekly GWG, especially PE. Above the weekly GWG optimal range, the incidence of PE increased; in contrast, the risk of PE was reduced. Obesity is a high-risk factor for PE by causing an inflammatory response to adipose tissue. Insufficient weight gain decreases the incidence of preeclampsia by reducing adipose tissue, thereby reducing inflammation^16,17^. This result is consistent with previous studies^16,18–20^. The rates of GDM were higher for women with lower weekly GWG in all BMI groups, and the incidence of GDM was significantly different in the other three groups except the obese group. Our study supports prior investigations that rates of GDM were higher in women with normal weight and GWG below IOM guidelines^21^. Similarly, Li-hua Lin’s paper showed that GWG below the optimal range increases the risk of GDM in the 3rd trimester^22^. A previous study found that excessive GWG in the first trimester and insufficient weight gain in the second trimester increased the risk of GDM in singleton pregnancy^23,24^. Additionally, Ping Y’s study found that excessive GWG before diagnosis increases the incidence of diabetes and diabetes-related complications in singleton pregnancy^25^. When GDM is diagnosed, nutrition control leads to slower weight gain in the later period, which may affect the total GWG. This may be one of the reasons for our results which are inconsistent with clinical observations. A preliminary study found that GWG is not linear^22^. Weekly GWG can only reflect the total GWG level to compare the overall GWG of different gestational weeks and cannot reflect the trajectory of GWG. Then, we need to further probe the GWG trajectories of twin pregnancies to clarify the relationship between GWG and GDM in twin pregnancies. Insufficient GWG increased the incidence of PROM and PB < 36 weeks. The result for PB was consistent with reports by Gold Stein et al. and Kominiarek et al.^22,26^. The result for PROM was consistent with the underlying mechanism of increased PROM, which may be due to nutritional deficiencies, especially the lack of copper, zinc and vitamins, which affect the synthesis of collagen fibers and elastic fibers of the fetal membrane and decrease the tensile capacity of the fetal membrane, which easily cause PROM^27^. Although there was no difference in the incidence of FGR among all the groups, it was still seen that increasing GWG had a tendency to increase fetal weight, which was similar to the results of previous studies^14^.

To our knowledge, this study is the first to calculate optimal GWG in a Chinese population of twins with optimal outcomes, and the participants underwent follow-up and delivery at the same institution, which is the largest center for ART, one of the largest maternal and child health care centers, and the largest twin birth center in Southwest China. The institution's intake of pregnant women was diverse, so the study population was representative. Another strength is that the study reversely validated the results by comparing maternal complication rates. However, there were still deficiencies in the article. This study is a retrospective study based on the data obtained from the discharge medical records, and we did not obtain the specific weight at each prenatal visit. When comparing the relationship between GWG and pregnancy outcomes, although the effect of gestational age on the overall weight during pregnancy was considered and weekly GWG was used as the research variable, due to the different speeds of weight gain in the first, second and third trimesters, weekly GWG was also limited. Therefore, there are no GWG optimal range for weight gain in the first, second, and third trimesters. Besides, since twin pregnant women in the obese group were very limited, it was also difficult to propose an optimal GWG range for obese women. Lastly, although we presented a GWG optimal range based on a Chinese population, due to the single-center design, the generalizability of this GWG optimal range was limited, an external validation process is required in other regions or centers.

In conclusion, we provide preliminary Chinese GWG optimal range which derived from twin-pregnant women with optimal outcomes (16–21.5 kg for underweight, 15–21.1 kg for normal weight, 13–20 kg for overweight), except for obesity, due to the limited sample size. Future prospective studies should be implemented to determine whether controlling pregnancy weight within the optimal range can improve pregnancy outcomes based on prepregnancy BMI and the trajectory of weight gain during pregnancy in different periods.

## Methods

### Participant selection

This retrospective study was conducted in Women and children’s Hospital of Chongqing Medical University, one of the largest childbirth hospitals in Southwest China. Data studied in this paper were obtained from electronic medical records between January 1, 2017, and December 31, 2021. Information was recorded for each case of twin pregnancies for the following information: maternal age, past medical history, and delivery history; pregnancy complications; maternal prepregnancy weight, height, gestational age at birth; birthweight; and neonatal outcomes. The following cases were excluded: (1) incomplete information; (2) gestational age at birth < 240/7 weeks; (3) triplet pregnancy; (4) monoamniotic twins; (5) monochorionic diamniotic twins complicated by twin anemia polycythemia sequence or twin-to-twin transfusion syndrome; and (6) reduction or stillbirth of one or two fetuses.

### Ethics statements

The study was reviewed and approved by the Ethics Committee of Chongqing Health Center for Women and Children (protocol number: (2021)伦审(科)026号). Written informed consent for participation was waived by the Ethics Committee of Chongqing Health Center for Women and Children due to retrospective study design. We declare that all methods were conducted in agreement with the relevant regulations and guidelines.

### Definitions and protocols

All pregnant women who gave antenatal care in our hospital were asked to provide their weight, including their prepregnancy weight, and to conduct measurements of height in the Obstetrical Outpatient Clinic. Pregnant women were asked to complete their antenatal care in 2- to 3-week intervals until 28 weeks, 2-week intervals until 34 weeks, and then weekly until birth. At each visit, blood pressure and maternal weight were measured and recorded in the individual’s antenatal care handbook. After hospitalization, height and prepregnancy weight were self-reported by pregnant women, and weight prior to delivery was measured. All the maternal baseline data and pregnancy outcomes were collected from medical records.

Gestational age was verified by crown-rump length through B-ultrasound in the first trimester. If the pregnancy was caused by the result of in vitro fertilization-embryo transplantation, gestational age was confirmed from the time of embryo transfer. Prepregnancy BMI was defined as prepregnancy weight (in kilograms) divided by squared height (in meters). The participants were classified into four groups according to the Chinese BMI standard: underweight (< 18.5 kg/m^2^), normal weight (18.5–23.9 kg/m^2^), overweight (24–27.9 kg/m^2^), and obese (≥ 28 kg/m^2^)^10,15^. Total GWG as the primary variable was computed as weight before delivery minus prepregnancy weight. In addition, GWG is related to the duration of gestation, and we calculated the weekly GWG (in kg/wk) by dividing the total GWG (in kilograms) by gestational age (in weeks) at birth.

Chorionicity was identified through ultrasonic examination to determine the fetal membrane and placenta insertion point is "twin peak" or "T" word (the former is dichorionic, the latter is monochorionic) between 10 and 14 weeks of gestation^28,29^. GDM was diagnosed when one or more of the venous plasma glucose levels exceeded the following cut-offs: fasting plasma glucose ≥ 5.1 mmol/L,1-h plasma glucose ≥ 10.0 mmol/L, 2-h plasma glucose ≥ 8.5 mmol/L via 2-h 75 g oral glucose tolerance test (OGTT)^30^. Gestational hypertension (GH) is defined as the first onset of hypertension after 20 weeks of gestation, systolic blood pressure ≥ 140 mmHg and/or diastolic blood pressure ≥ 90 mmHg, and a negative urine protein test^31^. Preeclampsia (PE) was defined as gestational hypertension accompanied by any of the following: 1) urinary protein quantification ≥ 0.3 g/24 h, or urinary protein/creatinine ratio ≥ 0.3, or random urinary protein ≥ ( +); No albuminuria, but accompanied by any of the following organs or systems involved: heart, lung, liver, kidney and other important organs, or abnormal changes in the blood system, digestive system, nervous system, placenta—fetal involvement, etc^31^. Premature rupture of membranes (PROM) was defined as natural rupture of membranes before regular contractions^32^. Newborns with birth weight below the 10th percentile of weight for gestational age are referred to as FGR. PB refers to those who give birth before 37 weeks gestation^33^.

### Determination of optimal gestational weight gain

Two steps were used to confirm the optimal GWG range in twin pregnancy in our population. To calculate the optimal GWG during pregnancy, we divided the participants into 2 subgroups (optimal outcome subgroup and adverse outcome subgroup). The optimal outcome subgroup needed to meet all of the following criteria: delivery gestational age ≥ 36 weeks, no pregnancy complications (GDM, GH, PE), birth weight of both twins ≥ 10th percentile, no twin-to-twin transfusion syndrome (TTTs), and no selective fetal growth restriction (sFGR). The adverse outcome subgroup was the population who did not meet these criteria.

The first step was proposing the optimal range of GWG using a statistical-based approach, which was identified by the distribution of GWG in the optimal outcome subgroup. The optimal range of GWG for women in different BMI categories was defined as the 25th to 75th centiles interquartile range (IQR) of GWG among women with optimal outcome in corresponding BMI group. The distribution of GWG in this optimal outcome subgroup was compared with the distribution of GWG in the adverse outcome subgroup.

The second step was confirming the proposed optimal range of GWG. We compared the incidence of pregnancy complications in the women below or above the proposed optimal GWG to women in the proposed optimal GWG and then the relationships between weekly GWG and risk for pregnancy complications was analyzed using multivariate logistic regression to validated the rationality of the proposed optimal GWG. It is worth noting that when the delivery gestational age was ≥ 36 weeks, PROM did not increase the adverse outcomes of pregnant women and newborns, so PROM was not considered one of the pregnancy complications in the excellent outcome subgroup. However, the age of PROM before 36 weeks increases the risk of maternal–fetal complications and is unfavorable for perinatal outcomes, so in our second approach, PROM was included in the observation of pregnancy complications.

### Statistical analysis

Means (± SDs) was presented for continuous variables, and the ratio (%) was used to present categorical variables. Comparisons of continuous variables were performed by One-Way analysis of variance and comparisons of categorical variables were performed by chi-squared tests or Fisher exact test. Baseline characteristics and outcomes between 4 BMI groups were compared. Following multivariate analysis, binary logistic regression was performed to identify the relationship between pregnancy complications and weekly GWG. Odds ratios (ORs) and 95% confidence intervals (CIs) were calculated. Covariates including age, height, prepregnancy weight and prepregnancy BMI.

Data were analysed using SPSS version 26.0 (IBM, Armonk, NY, USA). *P* < 0.05 was considered statistically significant.

## Data Availability

Data are available from the corresponding author upon reasonable request. All data are included in the article.
